# Molecular analysis of primary and metastatic sites in patients with renal cell carcinoma

**DOI:** 10.1172/JCI176230

**Published:** 2024-05-30

**Authors:** Shuchi Gulati, Pedro C. Barata, Andrew Elliott, Mehmet Asim Bilen, Earle F. Burgess, Toni K. Choueiri, Sourat Darabi, Nancy Ann Dawson, Benjamin Adam Gartrell, Hans J. Hammers, Elisabeth I. Heath, Daniel Magee, Arpit Rao, Charles J. Ryan, Przemyslaw Twardowski, Shuanzeng Wei, James Brugarolas, Tian Zhang, Matthew R. Zibelman, Chadi Nabhan, Rana R. McKay

**Affiliations:** 1 UC Davis Comprehensive Cancer Center, Sacramento, California, USA.; 2 University Hospitals Seidman Cancer Center, Cleveland, Ohio, USA.; 3 Caris Life Sciences, Irving, Texas, USA.; 4 Emory University School of Medicine, Atlanta, Georgia, USA.; 5 Levine Cancer Institute, Charlotte, North Carolina, USA.; 6 Dana Farber Cancer Institute, Boston, Massachusetts, USA.; 7 Hoag Memorial Hospital Presbyterian, Newport Beach, California, USA.; 8 Georgetown University Lombardi Comprehensive Cancer Center, Washington, DC, USA.; 9 Montefiore Medical Center and Albert Einstein College of Medicine, New York, New York, USA.; 10 UT Southwestern Medical Center, Dallas, Texas, USA.; 11 Karmanos Cancer Institute, Wayne State University School of Medicine, Detroit, Michigan, USA.; 12 Baylor College of Medicine, Houston, Texas, USA.; 13 University of Minnesota, Minneapolis, Minnesota, USA.; 14 Saint John’s Cancer Institute at Providence Saint John’s Health Center, Santa Monica, California, USA.; 15 Fox Chase Cancer Center, Philadelphia, Pennsylvania, USA.; 16 University of California San Diego, La Jolla, California, USA.

**Keywords:** Oncology, Cancer

## Abstract

**BACKGROUND:**

Metastases are the hallmark of lethal cancer, though underlying mechanisms that drive metastatic spread to specific organs remain poorly understood. Renal cell carcinoma (RCC) is known to have distinct sites of metastases, with lung, bone, liver, and lymph nodes being more common than brain, gastrointestinal tract, and endocrine glands. Previous studies have shown varying clinical behavior and prognosis associated with the site of metastatic spread; however, little is known about the molecular underpinnings that contribute to the differential outcomes observed by the site of metastasis.

**METHODS:**

We analyzed primary renal tumors and tumors derived from metastatic sites to comprehensively characterize genomic and transcriptomic features of tumor cells as well as to evaluate the tumor microenvironment at both sites.

**RESULTS:**

We included a total of 657 tumor samples (340 from the primary site [kidney] and 317 from various sites of metastasis). We show distinct genomic alterations, transcriptomic signatures, and immune and stromal tumor microenvironments across metastatic sites in a large cohort of patients with RCC.

**CONCLUSION:**

We demonstrate significant heterogeneity among primary tumors and metastatic sites and elucidate the complex interplay between tumor cells and the extrinsic tumor microenvironment that is vital for developing effective anticancer therapies.

## Abstract 2

Primary and distant metastatic sites in patients with renal cell carcinoma have distinct molecular features.

## Introduction

Renal cell carcinoma (RCC) is one of the most common cancers in the United States, affecting close to 80,000 patients annually (1). While most patients present with localized disease, up to 30% develop distant metastases after definitive resection, and a small fraction present with metastatic disease at diagnosis (2, 3). Metastasis is a multidirectional process, balanced between mitosis, “self-seeding” within the primary tumor, circulating tumor cells disseminating into circulation, and “seeding” of the distant site (4). Metastatic organotropism, the “seed and soil” hypothesis, (5) has been explored in several cancers as a complex phenomenon where metastatic spread from a primary tumor to metastatic sites is a nonrandom selective event driven by the characteristics of underlying cancer and the microenvironment at the metastatic site (6–8). As our understanding of cancer has evolved over the last few decades, we recognize that the interplay of tumor genomic and transcriptomic characteristics within the tumor microenvironment (TME), including immune cells, fibroblasts, endothelial cells, and other cell types (dependent on the site of metastasis), contribute to tumor behavior and potential therapeutic response (9).

RCC has a distinct and broad pattern of metastatic spread, with lung, bone, liver, and lymph node metastases being more common, while brain, endocrine glands, and the gastrointestinal tract metastases are less common (2, 10). In patients with RCC, there is heterogeneity in the disease’s natural history, dependent on the sites of metastases present. For example, liver, bone, and brain metastases portend inferior overall survival (OS), while glandular metastases, including those to the pancreas, lead to better outcomes (11–14). Investigators from the TRACERx cohort (15), have previously presented data on somatic alterations at primary and metastatic sites while examining metastasis-competent clones. We analyzed primary renal tumors and tumors derived from metastatic sites to comprehensively characterize genomic and transcriptomic features of tumor cells and evaluated the TME at both sites. We hypothesized that differential clinical behavior is likely driven by distinct molecular features of specific sites of metastasis. A better understanding of the molecular underpinnings and heterogeneity across sites of metastasis may ultimately pave a path for improved therapeutic targeting and combination treatments for patients with metastatic RCC.

## Results

### Patient cohort.

**Figure 1:**
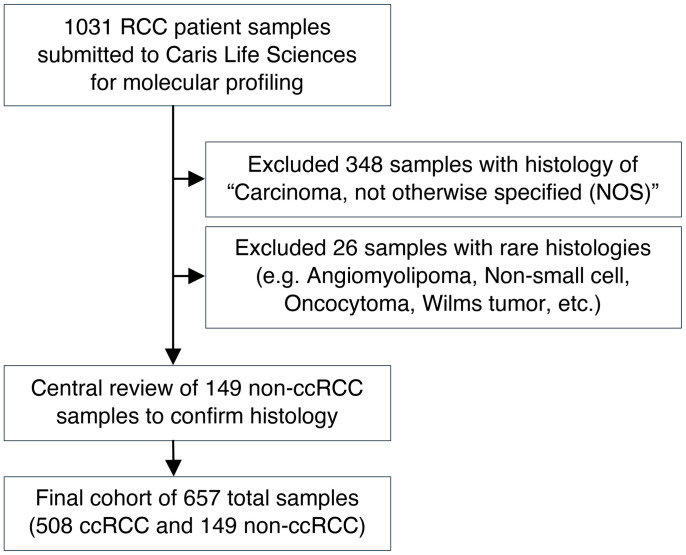
Consort diagram of study inclusion process.

**Figure 2:**
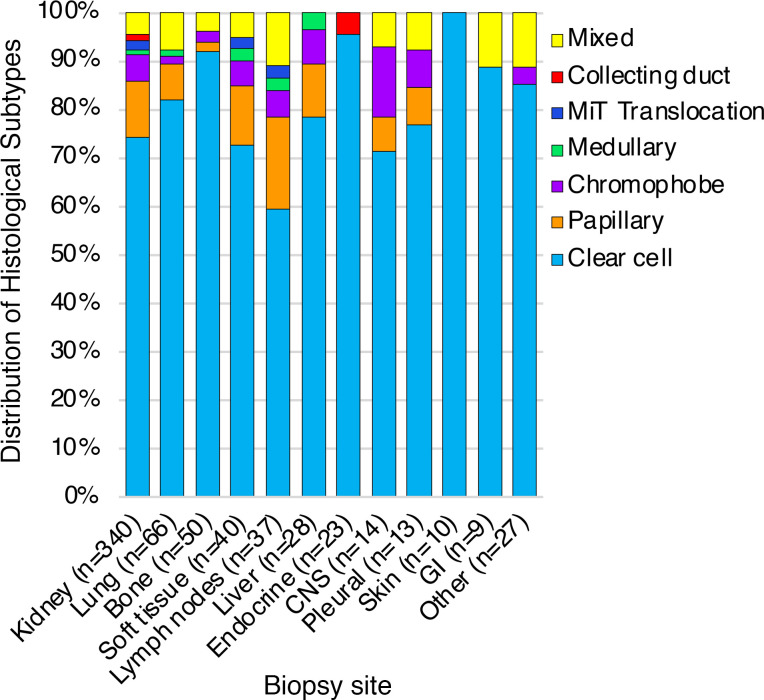
Distribution of histological subtypes by sites. Clear cell RCC is the most common histological subtype among all sites (primary as well as metastatic tumors). No difference was noted in histological subtype distribution by metastatic site relative to the kidney. GI, gastrointestinal; MiT, Microphthalmia transcription factor.

Formalin-fixed paraffin-embedded (FFPE) samples from patients with kidney cancer (*n* = 657) were submitted by clinical physicians to Caris Life Sciences, which is a commercial Clinical Laboratory Improvement Amendments of 1988–certified (CLIA-certified) laboratory for molecular profiling (Figure 1). All tumor samples annotated as nonclear-cell RCCs (non-ccRCCs) underwent central pathology review at Caris. Tumors classified as ‘other’ subtypes included samples with histologic features of more than 1 subtype, most commonly papillary with clear cell changes or unspecific features. Microphthalmia transcription factor (MiT) family translocation RCCs were confirmed by tumor genomic sequencing. We included a total of 657 tumor samples (derived from 654 patients) in this analysis; of which 340 (52%) tumors were from the primary site (kidney) and 317 (48%) tumors were from various sites of metastasis (Figure 2). Among metastatic sites, lung (*n* = 66; 10%), bone (*n* = 50; 7.6%), and soft tissue (*n* = 40; 6.1%) were the most common. Other sites included the liver (*n* = 28; 4.3%), endocrine organs (including adrenal glands, pancreas, and thyroid glands [*n* = 23; 3.5%]), and the central nervous system (CNS; *n* = 14; 2.1%). The median age of patients at tissue sampling was 62 years (range: 14–90+ years) and samples were collected from both male (*n* = 464; 70.6%) and female patients (*n* = 193; 29.4%) (Table 1). The most common histologic subtype was ccRCC, accounting for 508 (77%) of all tumors, followed by papillary (*n* = 63, 9.6%), and chromophobe (*n* = 30; 4.6%). Variant histologies including medullary, MiT translocation, and collecting duct carcinoma accounted for less than 5% of the total tumors. Sarcomatoid differentiation was observed in 62 (9.4%) tumors. There was no significant difference in histological subtype distribution by metastatic site relative to the kidney (*P* = 0.84) (Figure 2).

### Varying patterns of genomic alterations at primary kidney and metastatic sites.

**Figure 3:**
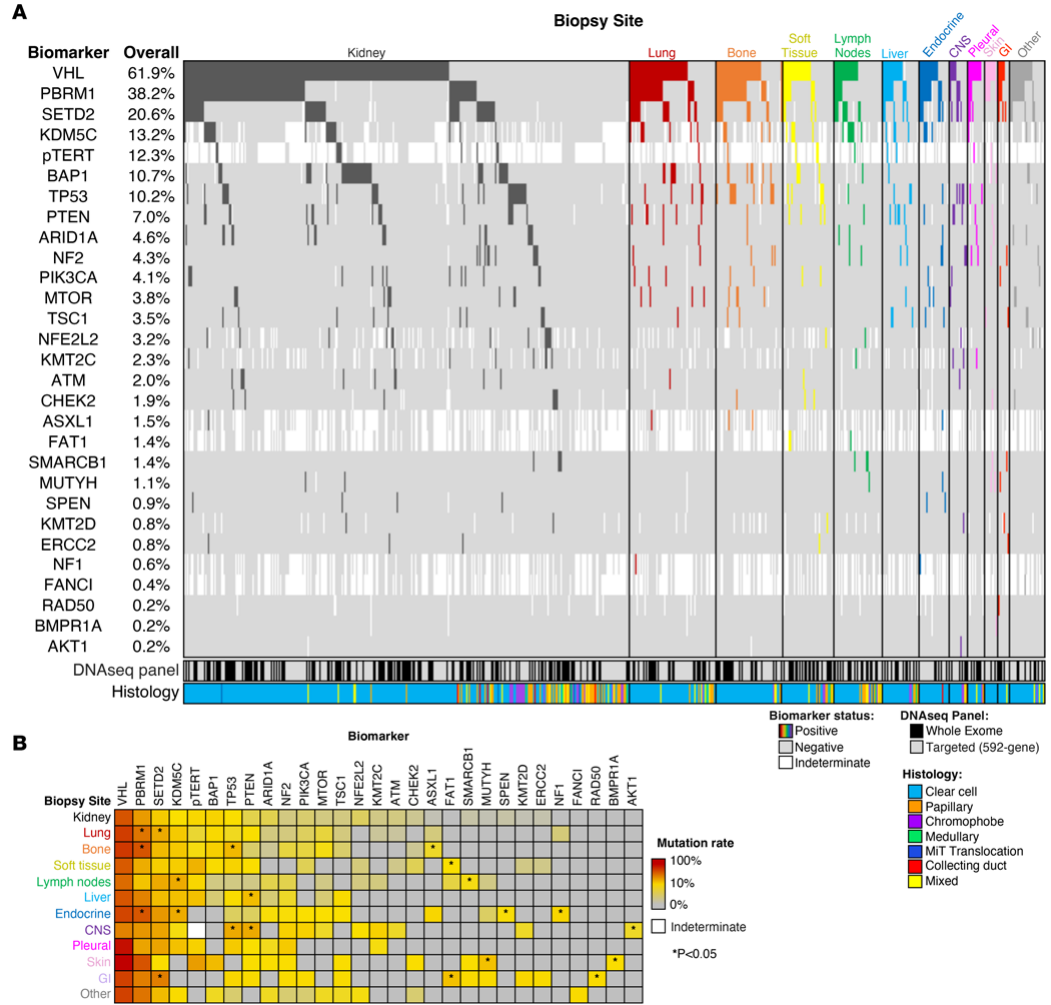
Somatic DNA alterations by site of patient tumor biopsy. (**A**) Oncoprint of the most common alterations (≥ 2% overall mutation rate), along with alterations present in the top 10 most frequently altered genes for any individual biopsy site. (**B**) Heatmap of mutation rates by metastatic site for genes shown in panel 2A. *P < 0.05. Abbreviations: CNS: central nervous system; GI, gastrointestinal; MiT, Microphthalmia transcription factor; pTERT, TERT promoter.

We performed an analysis of all somatic DNA alterations and identified 16 frequently mutated genes (with an overall prevalence of at least 2%) across all primary kidney and metastatic samples (Figure 3A). As reported in previous studies (16), the most frequently altered genes were von Hippel-Lindau (*VHL*) (61.9%, *n* = 406/656), Polybromo1 (*PBRM1*) (38.2%, *n* = 248/649), SET Domain Containing 2 (*SETD2*) (20.6%, *n* = 131/637), Lysine Demethylase 5C (*KDM5C*) (13.2%, *n* = 64/485) and BRCA1-associated protein-1 (*BAP1*) (10.7%, *n* = 68/633).

Compared with the primary renal tumor, patterns of genomic alterations varied among tumors derived from distinct metastatic sites (Figure 3B). For example, lung metastases were significantly enriched for *PBRM1* (47.7%, *n* = 31/65) and *SETD2* mutations (28.1%, *n* = 18/64); bone metastases were significantly enriched for *TP53* (22.9%, *n* = 11/48) mutations in addition to *PBRM1* (59.6%, *n* = 28/47), and *ASXL1* mutations (7.1%, *n* = 2/28); lymph node metastases were significantly enriched for *KDM5C* (28.0%, *n* = 7/25); liver metastases were enriched for phosphatase and tensin homolog (*PTEN*) mutations (21.4%, *n* = 6/28); endocrine gland metastases (including pancreas) were significantly enriched for *PBRM1* (59.1%, *n* = 13/22), *KDM5C* (27.8%, *n* = 5/18), *SPEN* (8.7%, *n* = 2/23), and *NF1* mutations (12.5%, *n* = 1/8), with limited tumor suppressor gene alterations. While tumors from other metastatic sites had fewer samples, additional alterations were identified, including soft tissue metastases enriched for *FAT1* (9.1%, *n* = 2/22) and CNS lesions significantly enriched for *TP53* (28.6%, *n* = 4/14), *PTEN* (28.6%, *n* = 4/14), and *AKT1* mutations (7.1%, *n* = 1/14) (each *P* < 0.05).

We performed a subanalyses only in non-clear cell tumors We noted that, after removing the non-clear tumors, the overall results for the clear cell-cohort did not change significantly (Supplemental Table 1A; supplemental material available online with this article; https://doi.org/10.1172/JCI176230DS1)). The most frequently altered genes in the primary renal lesions remained *VHL*, *PBRM1*, *SETD2*, *BAP1*, and *KDM5C* (Supplemental Table 1B). Results at metastatic sites from the pure ccRCC cohort remained similar to the overall cohort as well (Supplemental Table 1, C–M). The non-clear cohort (*n* = 149), had a distinct profile, in that the primary kidney tumors had the highest percentage of alterations in the *TERT* promoter (17.4%), *TP53* (13.1%), *SETD2* (11.6%), *VHL* (8%), and *ARID1A* (8%) (Supplemental Table 2A). Given smaller numbers belonging to each subgroup of metastatic sites, definitive conclusions were difficult to draw. However, similar to bone metastases from the entire cohort, nonclear-cell metastatic lesions from bone and the CNS were enriched for *TP53* mutations (Supplemental Table 2, B–M).

### The distribution of transcriptomic signatures varies between primary renal tumor and metastatic sites.

**Figure 4:**
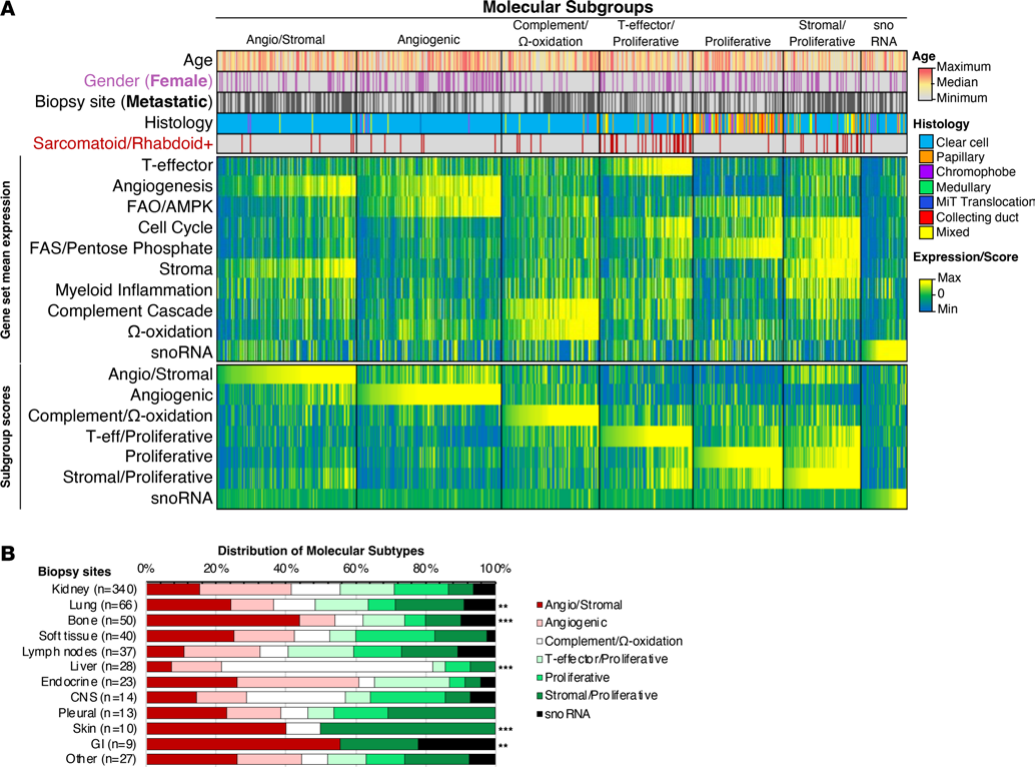
Sites of metastases have distinct distributions of molecular subtypes. (**A**) Heatmap of patient age, gender, tumor histology, average gene set expression levels, and molecular subgroup composite scores, with patient samples sorted in ascending order (left to right) of composite scores for each subgroup. (**B**) Distribution of molecular subtypes by organ site. **P < 0.01 and ***P < 0.001 when compared with kidney. FAO, fatty acid oxidation; AMPK, AMP-activated protein kinase; FAS, fatty acid synthesis; GI, gastrointestinal; MiT, Microphthalmia transcription factor.

We next evaluated samples based on the previously validated molecular subgroups derived from IMmotion151, a phase III trial investigating atezolizumab plus bevacizumab compared with sunitinib in patients with previously untreated metastatic ccRCC (17, 18) and, as detailed in the methods section, targeted somatic variant and integrated transcriptomic analysis on pretreatment tumor samples from this trial identified 7 distinct molecular clusters (17). We applied the cluster designations to our data set and assessed the distribution of molecular subtypes between kidney and metastatic samples (Figure 4A). Metastatic tumors were most frequent in the angiogenic/stromal (60.9%, *n* = 81/113) and stromal/proliferative (67.6%, *n* = 50/74) subgroups, with metastases comprising smaller proportion of the angiogenic (35.5%, *n* = 49/138), proliferative (38.3%, *n* = 33/86), and T-effector/proliferative (41.6%, *n* = 37/89) subgroups.

Next, we evaluated the distribution of molecular subtypes of distinct metastatic sites and noted significant differences across lung, bone, liver, skin, and GI tract metastases compared with the primary kidney (Figure 4B). Angiogenic/stromal and stromal/proliferative subgroups were enriched in lung (24.4%, *n* = 16; and 19.7%, *n* = 13, respectively, *P* < 0.01) and skin metastases (40.0%, *n* = 4; and 50.0%, *n* = 5, respectively, *P* < 0.001) compared with primary kidney tumors (15.3%, *n* = 52; and 7.1%, *n* = 24, respectively). Though nonsignificant (likely due to the limited sample size), greater proportions of angiogenic/stromal and angiogenic subtypes were also observed in endocrine metastases (26.1%, *n* = 6; and 34.8%, *n* = 8, respectively, *P* = 0.42). Bone and GI tract metastases were enriched with the angiogenic/stromal subtype (bone: 44.0%, *n* = 22, *P* < 0.001; GI: 55.6%, *n* = 5, *P* < 0.01), while liver metastases were enriched with the complement/Ω-oxidation subtype (60.7%, *n* = 17; *P* < 0.001) (Figure 4B).

In a subanalyses, where the nonclear-cell cohort was separated from the clear-cell cohort, we note that while ccRCC is largely driven by Angiogenesis and Stromal gene sets, whereas non-ccRCC is very low for Angiogenesis and largely driven by proliferative (fatty acid synthesis (FAS)/ pentose phosphate pathway). (Supplemental Table 3). When reviewing metastatic lesions from patients with non-ccRCC, lung lesions were similarly enriched for proliferative subtypes. Bone metastases were enriched more for the angiogenic/stromal subtype; however, bone metastatic lesions still had prominent proliferative and stromal/proliferative subtypes. The number of patients with non-ccRCC in each of the distinct metastatic site categories was small.

**Figure 5:**
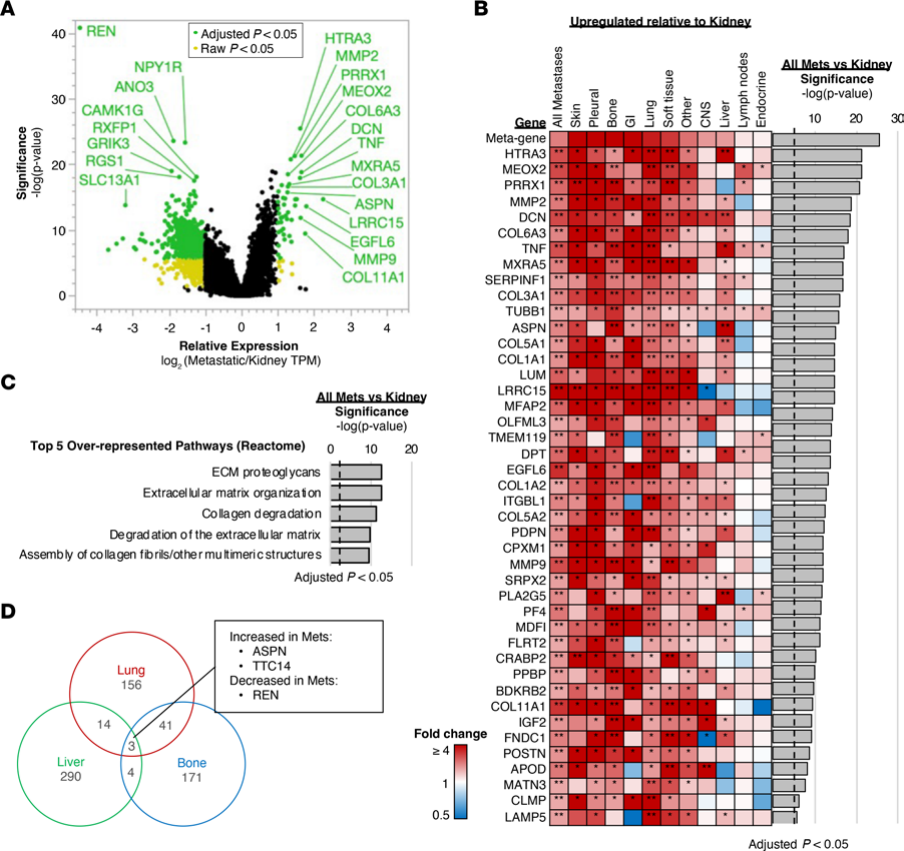
Gene expression profiling of metastatic and kidney biopsy sites. (**A**) Volcano plot of relative gene expression of all metastatic samples compared with kidney. Colored data points indicate log_2_ (fold changes) ≥ 1 or ≤ –1 and raw (yellow) or adjusted (green) *P* value < 0.05. (**B**) Expression of genes significantly upregulated in panel **A** for each metastatic site relative to kidney. *Raw *P* < 0.05, **Adjusted *P* < 0.05. (**C**) Pathway analysis of the significantly upregulated genes in panel **A**, with the top 5 over-represented pathways from Reactome database shown. (**D**) Venn diagram representing differentially expressed genes for lung, bone, and liver sites compared with kidney. met, metastases; GI, gastrointestinal.

We then assessed the differential gene expression profiles of primary kidney tumors compared with all metastatic sites (Figure 5). Among many genes with increased expression in kidney tumors, the *REN* gene that encodes renin, an aspartic protease that is secreted by the kidneys and drives the renin-angiotensin-aldosterone system involved in the regulation of blood pressure and electrolyte balance, was the most significantly upregulated gene in primary tumors compared with metastases (Figure 5A). In contrast, a total of 43 genes were identified with significantly increased expression in metastases (≥ 2-fold compared with kidney tumors), including upregulation of genes involved in extracellular matrix (ECM) reorganization, including collagen/proteoglycan assembly (i.e., *ASPN*, *DCN*, *COL6A3*, and *COL11A1*) and disassembly processes (i.e., *HTRA3*, *MMP2*, and *MMP6*) in most metastatic sites, with the exception of lymph node and endocrine metastases (Figure 5B). Pathway analyses of the genes upregulated in metastases further supported the enrichment of genes in ECM reorganization and related pathways (Figure 5C). Overlap of differentially expressed genes (specifically, those significantly different from the kidney) for bone, liver, and lung metastases was limited, with only *ASPN* and *TTC14* significantly upregulated and *REN* significantly downregulated for each site (Figure 5D). Of note, *ASPN*, a small leucine-rich proteoglycan expressed predominantly by cancer-associated fibroblasts (CAFs), plays a pivotal role in tumor progression by playing a role in modulating the TME (19, 20).

We also performed separate analyses for the median expression of top differentially expressed genes (DEGs) in the primary kidney versus all metastatic sites among clear-cell and non-clear cell cohorts. We found that the genes that we previously found to be associated with metastases hold true for both ccRCC and non-ccRCC samples when analyzed separately. (Supplemental Table 4 and Supplemental Figure 1).

### TMEs of distant metastases are distinct from primary kidney tumors.

**Figure 6:**
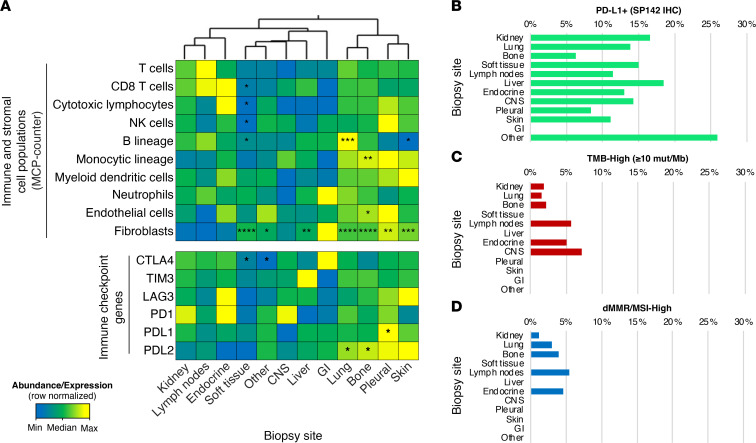
TME and immunotherapy-associated predictive biomarkers by site of metastases. (**A**) Heatmap of median cell abundance and gene expression by biopsy site. *P < 0.05, **P < 0.01, ***P < 0.001, and ****P < 0.0001 when compared with kidney. Frequency of biomarker-positive samples for (**B**) PDL1 IHC (SP142 antibody), (**C**) TMB-High (≥ 10 mut/Mb), and (**D**) dMMR/MSI-High. NK, natural killer; CTLA4, The cytotoxic T-lymphocyte-associated antigen-4; TIM3, T cell immunoglobulin domain and mucin domain3; LAG3, Lymphocyte-Activation Gene3; PD-1, programmed death-1; PDL1, programmed death ligand-1; PDL2, programmed death ligand-2; GI, gastrointestinal.

Within the TME, there are a variety of mechanisms by which cancer cells interact with the noncancerous host cells. These intercellular interactions include those that occur via cell-cell contact and others that occur via paracrine signaling mediated by cytokines, chemokines, and growth factors (21). To better understand the TME contribution to metastatic behavior in RCC, we estimated the abundance of cell populations in the TME, noting significant differences across metastatic sites relative to the primary renal tumor. Endothelial cells and cells of monocytic lineage were more abundant in bone metastatic lesions while lung and skin metastasis had an abundance of B-lineage cells. Although not significant, there was a greater abundance of T cells among lymph node metastases and a greater abundance of CD8^+^ and cytotoxic T cells in endocrine metastases compared with the kidney. In general, fibroblast cell populations were more abundant across metastatic sites, especially among liver, lung, bone, pleural, and soft tissue metastatic sites (Figure 6A). While the RNA expression of immune checkpoint genes such as *CTLA4*, *TIM3*, *LAG3*, and *PD1*, was not significantly different between the kidney and sites of metastasis, *PDL1* expression was higher in the pleura and *PDL2* expression was higher in the lung and bone metastases than in the kidney.

Lastly, we evaluated the frequency of putative biomarkers of response to immune-checkpoint inhibitors at metastatic sites, including PD-L1^+^ expression by IHC (SP142 antibody), high tumor mutation burden (TMB-High ≥ 10 mutations/ Mb), and mismatch repair protein deficiency/high microsatellite instability (dMMR/MSI-high) status. Biomarker frequencies were not significantly different across metastatic sites relative to the kidney (Figure 6, B, C, and D).

Consistent with the analysis previously discussed, which demonstrated low representation of non-ccRCC in the T-effector subgroup, non-ccRCC are relatively “cold” compared to ccRCC, with a lower infiltrate of CD8^+^ T and cytotoxic T cells. (Supplemental Table 5, A and B, and Supplemental Table 6).

## Discussion

This study presents comprehensive molecular profiling of a large set of RCC tumors from the primary kidney and metastatic sites to delineate differences that may contribute to clinical behavior. Previous studies have elucidated that site of metastasis informs prognosis in RCC. While patients with liver, bone, and brain metastases tend to have the worst outcomes, those with metastases to endocrine glands including the pancreas have improved outcomes (11, 13–15). The TRACERx Renal cohort identified unique somatic alterations in primary and metastatic tumors and was able to classify groups of tumor clones based on their ability to metastasize (15). Other studies have identified a higher number of genetic aberrations associated with metastatic lesions when compared with primary lesions and have described significant intratumoral heterogeneity even from samples collected from the same site (22–24). Another smaller cohort study specifically described angiogenesis, cell migration, cell motility, and cell adhesion–related gene signatures to be associated with pulmonary metastases in patients with RCC (25). MicroRNAs, as they are involved in the control of cell development, proliferation, and apoptosis and thus promotion of metastatic spread, have also been described (26–28). Biologically, earlier studies have elucidated the genomic landscape of metastatic lesions related to RCC; we add to this body of literature by presenting data from a real-world cohort. We also add detailed transcriptomic data and investigated the TME, which are lacking in previously published data sets. This work is clinically relevant and can inform strategies for optimizing therapy for patients with advanced RCC.

We first identified differential patterns of genomic alterations across kidney and metastatic sites. For example, metastatic lesions from the lungs, endocrine glands, and bone lesions were enriched for *PBRM1* mutations relative to the kidney (*P* < 0.05 at each site). Retrospective data reveal that patients with lung and pancreatic metastases (included in our analysis with “endocrine”) are associated with better clinical outcomes, attributable to a preponderance of *PBRM1* mutations in the latter (29). Bone lesions also had higher *TP53* alterations, which are associated with negative outcomes and may supersede the benefits associated with *PBRM1* mutations. As far as the role of *PBRM1* mutations in predicting response to treatments, there have been discrepancies in results across different data sets regarding the predictive role of *PBRM1* mutations. While in some contexts, *PBRM1* mutated tumors expressed high levels of angiogenesis-associated genes and were associated with better prognosis in patients treated with antiangiogenic drug-based regimens (30), in other studies patients with *PBRM1* mutations had better clinical outcomes in response to immune checkpoint inhibitors (31, 32). Due to lack of outcome data in our study, we cannot verify this association, but we did see a predominance of *PBRM1* mutations in pancreatic and bone metastatic lesions that have previously been shown to respond better to antiangiogenic agents (29, 33).

*BAP-1* mutations, which have been associated with poor prognosis in ccRCC (34, 35), were observed in 10% of tumors in the overall cohort, consistent with prior studies (16, 36–38), and no differential expression was seen between the primary renal tumors versus sites of metastasis.

We observed *PTEN* alterations in 7% of tumors in our cohort with increased alteration in tumors derived from the liver and CNS. *PTEN* mutations in ccRCC cell lines have been shown to promote sensitivity to mTOR inhibitors, everolimus, and temsirolimus (39). In this study, although rates of tumors with *PTEN* loss were low, we found metastatic lesions, particularly liver metastases, enriched for *PTEN* mutations, suggesting that further study of mTOR-targeting agents in patients with liver metastasis may be warranted.

Another important mutation with differential prevalence between primary renal tumors and metastatic sites was *TP53*, a classic tumor suppressor gene. Prior studies demonstrate *TP53* to be prevalent in approximately 2%–6 % of tumors at primary sites versus up to 15% at metastatic sites (16, 38, 40–42). Additionally, *TP53* mutations have been associated with poor outcomes (43). Our observation of a higher prevalence of *TP53* mutations in bone and brain lesions is consistent with the observation that these sites of metastasis are associated with worse clinical outcomes (14).

Current treatment options in the frontline setting include immunotherapy combinations (nivolumab plus ipilimumab) or the combination of a checkpoint inhibitor with a VEGF inhibitor. Transcriptomic analysis in our study recapitulates data from IMMotion151, which identified 7 molecular clusters in RCC: (1) angiogenic/stromal; (2) angiogenic; (3) complement/Ω-oxidation; (4) T-effector/proliferative; (5) proliferative; (6) stromal/proliferative, and (7) small nuclear RNA (snoRNA) genes. We observed that metastatic tumors from the lungs were enriched with angiogenic/stromal and stromal/proliferative subtypes, while bone metastasis had a predominance of angiogenic/stromal subtypes, suggesting that these patients may respond to an antiangiogenic drugs. Though prospective data of outcomes of patients with bone metastases treated with pure IO or IO/VEGF regimen are lacking, retrospectively, tyrosine kinase inhibitor–containing (TKI-containing) regimens compared with immunotherapy alone have been shown to improve outcomes for patients with bone metastases (33).

In our study, liver lesions were uniquely and significantly enriched for genes belonging to the complement/Ω-oxidation (cluster iii). The “complement/Ω-oxidation cluster” has previously been shown to have a low expression of both angiogenesis and immune genes (17). Moderate expression of cell cycle genes has been associated with elevated expression of genes belonging to the complement cascade signature, which are known to drive worse outcomes (17). As shown above, liver metastatic lesions are also enriched for *PTEN* mutations, which have been implicated in resistance to the antiangiogenic agents sunitinib and sorafenib (44) and a higher responsiveness to mTOR inhibitors such as everolimus and temsirolimus. While rarely used as a single agent, consideration for the combination of lenvatinib and everolimus is provocative for patients with liver metastases.

While uncommon in our study, CNS metastases had a numerically higher percentage of T-effector, proliferative, and stromal/proliferative subtypes compared with the kidney, suggesting potential benefit of immune checkpoint inhibition for patients with CNS metastases. A recent retrospective study from the IMDC database also showed that patients with brain metastases receiving IO-based combination therapy had longer OS than those receiving anti-VEGF monotherapy (HR 0.51, 95% CI 0.29–0.92; *P* = 0.026) (45).

Many of the same genes that were identified as associated with metastases in our study were reported as associated with metastases in studies that used data from the TCGA or other publicly available data sets (46, 47), including genes involved in ECM reorganization. In addition to the interaction between cancer cells and the TME, there are paracrine effects from the release of cytokines, chemokines, growth factors, and proteases which determine behavior of tumors at metastatic sites (21). Based on our analysis, we found a predominance of fibroblasts across several metastatic sites (lung, pleura, bone, liver, skin, and soft tissue). CAFs have been proposed to play an immune regulatory/inflammatory function as well as an antigen-presenting function (47). Previously, in lung metastasis from breast cancer, CAFs were described to be transcriptionally dynamic with stage-specific gene signatures and have been implicated in shaping the inflammatory microenvironment in these lesions (48). Proinflammatory signaling from CAFs (IL-33) has also been implicated in establishing a metastatic niche in the context of breast cancer–associated lung metastasis and chemoresistance (49, 50). When compared with the kidney, markers of ICI response such as PD-1, dMMR/MSI-High, and high TMB were similar at distant sites of metastasis. Even though previous studies have shown a discordance as high as approximately 21% in PD-L1 staining between primary tumors and metastatic sites in RCC (51), our study did not show this difference. However, our study did not use paired samples. Interestingly, transcriptional expression of PD-L1 and/or PD-L2, which have been shown to be associated with worse prognosis (14), were higher in lung, pleura, and bone metastases compared with kidney samples. A previous metaanalysis of more than 1,000 cases with RCC demonstrated higher PD-L1 expression by IHC to be associated with an increase in mortality by over 50% (52). However, PD-L1 expression has not consistently predicted response to immune checkpoint inhibition in several clinical trials (53). This is further complicated by differences between PD-L1 assays by IHC that are used for each drug (e.g., 28–8 Dako assay for nivolumab, and SP142 Ventana assay for atezolizumab) (54). These factors have discouraged the use of PD-L1 as a predictive biomarker of response to immune checkpoint inhibitor therapy for patients with RCC.

Overall, this study provides the rationale for potential clinical activity of treatments under development in patients with specific metastatic sites. To our knowledge, an in-depth comparative analysis of primary and metastatic lesions from both a genomic and transcriptomic perspective and that of the TME in a large RCC cohort has not been presented before. We provide new insights to understand the molecular underpinning of organotropism, which will help inform future personalized therapy strategies in patients with RCC. However, our study is limited by the lack of clinical and survival data to correlate molecular associations of metastases with outcomes, as well as evaluation of intrinsic and acquired resistance mechanisms. Moreover, while we assume most tumors included in this analysis are from patients with metastatic disease, the precise stage information for individual tumors was not available. Additionally, we lack a comparison of paired primary and metastatic samples derived from the same individual patient to assess tumor evolution. While central pathology review was conducted on all cases classified as non-ccRCC to confirm the diagnosis, this was not performed on all ccRCC cases. The tumors used in this analysis were FFPE and we do acknowledge that formalin fixation can chemically alter DNA/ RNA, and can affect our data analysis (55). However, for commercial next-generation sequencing, this is the most commonly utilized technique in clinical practice and our study was a retrospective evaluation of tumors previously submitted. Overall, our results help inform future precision medicine strategies for patients with RCC and to understand the potential of individualizing treatments according to specific sites of metastasis in the future.

In conclusion, cancer mortality is almost exclusively related to the development of distant metastasis. Cancer metastasis is a complex phenomenon where cancer cells are trying to establish a niche in various organs. Many factors have been proposed as plausible mechanisms, such as mutations within the cancer cells, suppressive signals in the TME (56–59), features of each organ of metastasis (architecture, physiology, and resident cells — mesenchymal cells such as activated fibroblasts, pericytes, endothelial cells, and inflammatory cells) (59). Our findings define several such molecular features that differentiate primary and distant metastatic sites of disease in patients with RCC. Future studies should address the clinical outcomes correlated with molecular differences of metastases and validate these findings in a clinical trial setting.

## Methods

### Sex as a biological variable.

Samples from both males and females were involved in this research as the findings are applicable to both groups.

### Study cohort.

FFPE samples from patients with kidney cancer (*n* = 657) were submitted by clinical physicians to a commercial CLIA-certified (Caris Life Sciences) laboratory for molecular profiling. All tumor samples annotated as non-ccRCCs underwent central pathology review at Caris. Tumors classified as ‘other’ subtypes included samples with histologic features of more than 1 subtype, most commonly papillary with clear cell changes, or unspecific features. MiT family translocation RCCs were confirmed by tumor genomic sequencing.

### DNA next-generation sequencing.

Next-generation sequencing (NGS) was performed on isolated genomic DNA using the NextSeq platform (Illumina, Inc.) for 592 cancer-relevant genes (*n* = 375 samples) or the Illumina NovaSeq 6000 platform (Illumina, Inc.) for whole exome sequencing (WES) (*n* = 282 samples) (Supplemental Tables 7 and 8). Prior to molecular testing, tumor enrichment was achieved by harvesting targeted tissue using manual microdissection techniques. A custom-designed SureSelect XT assay was used to enrich exonic regions of 592 whole-gene targets (Agilent Technologies). All variants were detected with more than 99% confidence based on allele frequency and amplicon coverage, with an average sequencing depth of coverage of over 500 and an analytic sensitivity threshold of 5% established for variant calling. For WES, a hybrid pull-down panel of baits designed to enrich for 720 clinically relevant genes at high coverage and high read depth was used, along with another panel designed to enrich for more than 20,000 additional genes at lower depth, including a SNP backbone panel (Agilent Technologies) consisting of 200K SNPs from exonic regions and 50K from intronic regions, with a minimum of 17 SNPs per Mb of genome sequence. Segment sizes range from 2–6 Mb, depending on segment proximity to the centromeres or telomeres, although 99% of segments are ≥ 5Mb. The copy number of each exon was determined by calculating the average depth of the sample along with the sequencing depth of each exon and comparing this calculated result to a precalibrated value, with a positive threshold of at least 6 copies used to calculate copy number amplification prevalence. Genomic variants were classified by board-certified molecular geneticists according to criteria established by the American College of Medical Genetics and Genomics (ACMG). When assessing mutation frequencies of individual genes, ’pathogenic,’ and ‘likely pathogenic’ were counted as mutations while ‘benign’, ‘likely benign’, and ‘variants of unknown significance’ were excluded.

### RNA WTS and fusion detection.

Whole transcriptome sequencing *(*WTS)uses a hybrid-capture method to pull down the full transcriptome from FFPE tumor samples using the Agilent SureSelect Human All Exon V7 bait panel (Agilent Technologies) and the Illumina NovaSeq platform (Illumina, Inc.). FFPE specimens underwent pathology review to discern the percent tumor content and tumor size; a minimum of 10% tumor content in the area for microdissection was required to enable enrichment and extraction of tumor-specific RNA. Qiagen RNA FFPE tissue extraction kit was used for extraction, and the RNA quality and quantity were determined using the Agilent TapeStation. Biotinylated RNA baits were hybridized to the synthesized and purified cDNA targets, and the bait-target complexes were amplified in a postcapture PCR reaction. The resultant libraries were quantified and normalized, and the pooled libraries were denatured, diluted, and sequenced. Raw data was demultiplexed using the Illumina DRAGEN FFPE accelerator. FASTQ files were aligned with STAR aligner (Alex Dobin, release 2.7.4a github). A full 22,948-gene data set of expression data was produced by the Salmon, which provides fast and bias-aware quantification of transcript expression (53) BAM files from STAR aligner were further processed for RNA variants using a proprietary custom detection pipeline. The reference genome used was GRCh37/hg19, and analytical validation of this test demonstrated 97% or higher Positive Percent Agreement (PPA), 99% or higher Negative Percent Agreement (NPA), and 99% or higher Overall Percent Agreement (OPA) with a validated comparator method. Identified fusion transcripts were further evaluated to determine breakpoint positions and functional domains retained from fused genes.

### RNA expression analyses.

Molecular subgroups (*n* = 7) were established using previously described gene sets by Motzer et al. (17, 18). The previous subgroups described were from a treatment-naive set of 823 tumor samples (625 primary and 198 metastatic) collected from patients enrolled in the phase-III clinical trial, IMotion151. This cohort included 688 tumors of clear cell histology without a sarcomatoid component, 110 tumors of clear cell histology with any sarcomatoid component, 1 tumor of clear cell histology with unknown sarcomatoid component, and 24 tumors of nonclear cell histology were also included. Overall, molecular stratification of these 823 RCC was able to subclassify the tumors into 7 groups, each with biologically distinct transcriptomic characteristics. Cluster 1 (angiogenic/stromal) and 2 (angiogenic) tumors were described as highly angiogenic, as well as associated with the presence of endothelial cells. Cluster 1 tumors, in addition, and in contrast to those in cluster 2, also had higher stroma-specific expression genes as well (fibroblast-derived, and stroma-associated genes (*FAP*, *FN1*, *PostN*, and *MMP2*). Cluster 3 (Complement/Ω-oxidation) tumors had a relatively lower expression of both angiogenesis and immune genes, while they had a moderately high expression of cell-cycle genes, genes associated with the complement cascade and the cytochrome P450 family. Cluster 4 (T-effector/Proliferative) tumors were enriched for genes coding for cell-cycle transcription factors and for immunogenic T-effector, JAK/STAT and interferon-α and -γ gene expression pathways and were associated with the highest expression of PD-L1 IHC as well as the highest infiltration of adaptive and innate immune cells (CD8^+^, CD4^+^, and regulatory T cells, B cells, macrophages, and dendritic cells). Clusters 5 (Proliferative) and 6 (Stromal/Proliferative) showed enrichment of the myeloid inflammation genes and had lower T-effector gene signatures. Cluster 6 also showed high expression of the epithelial-mesenchymal transition (EMT) transcriptional signature module and enrichment of collagen- and fibroblast-associated stromal genes. Clusters 4, 5, and 6 also enriched for an anabolic metabolism transcriptomic profile, with higher expression of genes associated with fatty acid synthesis and the pentose phosphate pathway. Cluster 7 (snoRNA) was characterized by enrichment of expression of snoRNAs.

Gene expression values were log-transformed and standardized to z scores, and coefficients for each signature were established based on the median scores previously reported for the 7 molecular subgroups. For each sample, a composite score was derived for each molecular subgroup by a sum of weighted average z scores, in which a higher composite score reflected greater similarity to the previously reported average expression profile for a molecular subgroup. Samples were classified as a single molecular subgroup according to the highest composite score for each sample.

To assess the relative abundance of immune and stromal cell populations in the TME, gene expression values were analyzed using the Microenvironment Cell Populations (MCP) counter tool.

### Tumor mutational burden.

Tumor mutational burden (TMB) was measured by counting all non-synonymous missense, nonsense, in-frame insertion/deletion, and frameshift mutations found per tumor that had not been previously described as germline alterations in dbSNP151, Genome Aggregation Database (gnomAD) databases, or benign variants identified by Caris’s geneticists. A cutoff point of ≥ 10 mutations per megabase (mt/MB) was used based on the KEYNOTE-158 pembrolizumab trial (54).

### IHC.

IHC was performed on full FFPE sections of glass slides. Slides were stained using the Agilent DAKO Link 48 (Santa Clara) automated platform and staining techniques, per the manufacturer’s instructions, and were optimized and validated per CLIA/CAP and ISO requirements. Staining was scored for intensity (0 = no staining; 1+ = weak staining; 2+ = moderate staining; 3+ = strong staining) and staining percentage (0%–100%). PDL1 (SP142) staining of ≥ 2+ and ≥ 5% tumor cells was categorized as positive.

### Deficient mismatch repair/microsatellite instability high.

A combination of multiple test platforms was used to determine the MSI or MMR status of the tumors profiled, including IHC (MLH1, M1 antibody; MSH2, G2191129 antibody; MSH6, 44 anti-body; and PMS2, EPR3947 antibody (Ventana Medical Systems, Inc.), fragment analysis (FA, Promega), and NGS (> 2800 target microsatellite loci were examined and compared to the reference genome hg19 from the UCSC Genome Browser database). The platforms generated highly concordant results as previously reported (Vanderwalde 2018 Cancer Medicine) and in the rare cases of discordant results, dMMR/MSI-High status was determined by the IHC results.

### Statistics.

All statistical analyses were performed with JMP V13.2.1 (SAS Institute) or R Version 3.6.1 (https://www.R-project.org). Continuous data were assessed using Mann-Whitney U test, and categorical data was evaluated using Chi-square or Fisher’s exact test, where appropriate.

### Study approval.

The present study was conducted in accordance with the guidelines of the Declaration of Helsinki, Belmont Report, and US Common Rule. With compliance to policy 45 CFR 46.101(b), this study was conducted using retrospective, deidentified clinical data, and patient consent was not required.

### Data availability.

The data sets generated during and/or analyzed during the current study (including the figures in the manuscript, supplemental files, and the supporting data value file) are available from the corresponding author upon reasonable request. The deidentified sequencing data are owned by Caris Life Sciences, and qualified researchers can apply for access to these summarized data by contacting Andrew Elliott and signing a data usage agreement.

## Author contributions

SG was responsible for designing research studies, conducting experiments, acquiring data, analyzing data, and writing the manuscript. PCB was responsible for designing research studies, conducting experiments, acquiring data, analyzing data, and writing the manuscript. AE was responsible for designing research studies, conducting experiments, acquiring data, analyzing data, and writing the manuscript. MAB was responsible for acquiring data and writing the manuscript. EFB was responsible for acquiring data and writing the manuscript. TKC was responsible for acquiring data, designing research studies, and writing the manuscript. SD was responsible for acquiring data and writing the manuscript. NAD was responsible for acquiring data and writing the manuscript. BAG was responsible for acquiring data and writing the manuscript. HJH was responsible for acquiring data and writing the manuscript. EIH was responsible for acquiring data and writing the manuscript. DM was responsible for acquiring data and writing the manuscript. AR was responsible for acquiring data and writing the manuscript. CJR was responsible for acquiring data and writing the manuscript. PT was responsible for acquiring data and writing the manuscript. SW was responsible for analyzing data, acquiring data, and writing the manuscript. JB was responsible for acquiring data and writing the manuscript. TZ was responsible for acquiring data and writing the manuscript. MRZ was responsible for acquiring data and writing the manuscript. CN was responsible for designing research studies, acquiring data, and writing the manuscript. RRM was responsible for designing research studies, conducting experiments, acquiring data, analyzing data, and writing the manuscript.

## Supplementary Material

Supplemental data

ICMJE disclosure forms

Supplemental tables 1-8

Supporting data values

## Figures and Tables

**Table 1:**
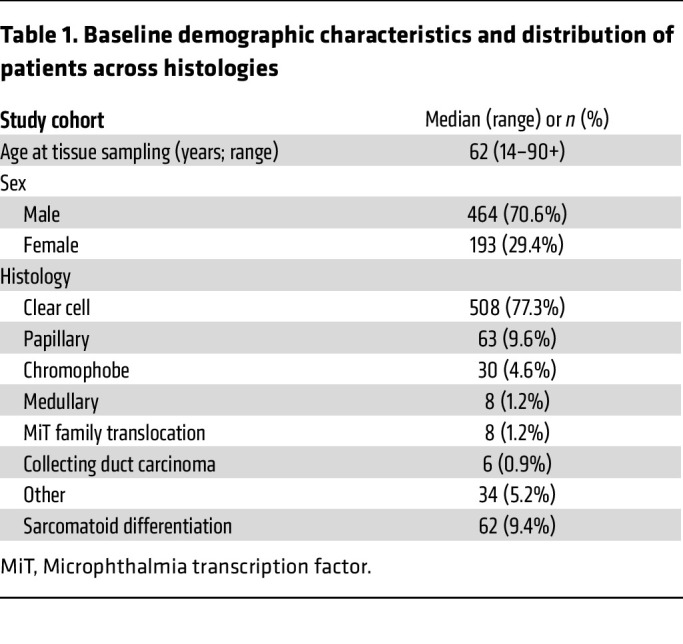
Baseline demographic characteristics and distribution of patients across histologies

## Version 1 07/15/2024

Electronic publication

## References

[1] https://seer.cancer.gov/statfacts/html/all.html.

[2] Gong J (2016). Metastasis in renal cell carcinoma: biology and implications for therapy. Asian J Urol.

[3] Wood E (2020). Adjuvant therapy for localized high-risk renal cell carcinoma. Urol Clin North Am.

[4] Comen E (2011). Clinical implications of cancer self-seeding. Nat Rev Clin Oncol.

[5] Paget S (1989). The distribution of secondary growths in cancer of the breast. 1889. Cancer Metastasis Rev.

[6] Chaffer CL, Weinberg RA (2011). A perspective on cancer cell metastasis. Science.

[7] Chen W (2018). Organotropism: new insights into molecular mechanisms of breast cancer metastasis. NPJ Precis Oncol.

[8] Liu Y, Cao X (2016). Characteristics and significance of the pre-metastatic niche. Cancer Cell.

[9] Bejarano L (2021). Therapeutic targeting of the tumor microenvironment. Cancer Discov.

[10] Bianchi M (2012). Distribution of metastatic sites in renal cell carcinoma: a population-based analysis. Ann Oncol.

[11] McKay RR (2014). Impact of bone and liver metastases on patients with renal cell carcinoma treated with targeted therapy. Eur Urol.

[12] Vickers MM (2013). Prognostic factors of survival for patients with metastatic renal cell carcinoma with brain metastases treated with targeted therapy: results from the international metastatic renal cell carcinoma database consortium. Clin Genitourin Cancer.

[13] Shaya JA (2021). Prognostic significance of pancreatic metastases in patients with advanced renal cell carcinoma treated with systemic therapy. Clin Genitourin Cancer.

[14] Dudani S (2021). Evaluation of clear cell, papillary, and chromophobe renal cell carcinoma metastasis sites and association with survival. JAMA Netw Open.

[15] Turajlic S (2018). Tracking cancer evolution reveals constrained routes to metastases: TRACERx Renal. Cell.

[16] Creighton CJ (2013). Comprehensive molecular characterization of clear cell renal cell carcinoma. Nature.

[17] Motzer RJ (2020). Molecular subsets in renal cancer determine outcome to checkpoint and angiogenesis blockade. Cancer Cell.

[18] Rini BI (2019). Atezolizumab plus bevacizumab versus sunitinib in patients with previously untreated metastatic renal cell carcinoma (IMmotion151): a multicentre, open-label, phase 3, randomised controlled trial. Lancet.

[19] Hughes RM (2019). Asporin restricts mesenchymal stromal cell differentiation, alters the tumor microenvironment, and drives metastatic progression. Cancer Res.

[20] Sasaki Y (2021). Expression of asporin reprograms cancer cells to acquire resistance to oxidative stress. Cancer Sci.

[21] Visser KE de, Joyce JA (2023). The evolving tumor microenvironment: From cancer initiation to metastatic outgrowth. Cancer Cell.

[22] Gerlinger M (2012). Intratumor heterogeneity and branched evolution revealed by multiregion sequencing. N Engl J Med.

[23] Bissig H (1999). Evaluation of the clonal relationship between primary and metastatic renal cell carcinoma by comparative genomic hybridization. Am J Pathol.

[24] Maruschke M (2014). Expression profiling of metastatic renal cell carcinoma using gene set enrichment analysis. Int J Urol.

[25] Wuttig D (2009). Gene signatures of pulmonary metastases of renal cell carcinoma reflect the disease-free interval and the number of metastases per patient. Int J Cancer.

[26] Heinzelmann J (2014). MicroRNAs with prognostic potential for metastasis in clear cell renal cell carcinoma: a comparison of primary tumors and distant metastases. Ann Surg Oncol.

[27] White NMA (2011). miRNA profiling in metastatic renal cell carcinoma reveals a tumour-suppressor effect for miR-215. Br J Cancer.

[28] Wotschofsky Z (2012). Identification of metastamirs as metastasis-associated microRNAs in clear cell renal cell carcinomas. Int J Biol Sci.

[29] Singla N (2020). Pancreatic tropism of metastatic renal cell carcinoma. JCI Insight.

[30] McDermott DF (2018). Clinical activity and molecular correlates of response to atezolizumab alone or in combination with bevacizumab versus sunitinib in renal cell carcinoma. Nat Med.

[31] Braun DA (2019). Clinical validation of PBRM1 alterations as a marker of immune checkpoint inhibitor response in renal cell carcinoma. JAMA Oncol.

[32] Miao D (2018). Genomic correlates of response to immune checkpoint therapies in clear cell renal cell carcinoma. Science.

[33] Challapalli A (2022). 1463P Patterns of care and outcomes of metastatic renal cell carcinoma (mRCC) patients (pts) with bone metastases (BM): A UK multicenter review. Ann Oncol.

[34] Gulati S (2022). BRCA1-associated protein 1 (BAP-1) as a prognostic and predictive biomarker in clear cell renal cell carcinoma: a systematic review. Kidney Cancer.

[35] Kapur P (2013). Effects on survival of BAP1 and PBRM1 mutations in sporadic clear-cell renal-cell carcinoma: a retrospective analysis with independent validation. Lancet Oncol.

[36] Dalgliesh GL (2010). Systematic sequencing of renal carcinoma reveals inactivation of histone modifying genes. Nature.

[37] Peña-Llopis S (2012). BAP1 loss defines a new class of renal cell carcinoma. Nat Genet.

[38] Sato Y (2013). Integrated molecular analysis of clear-cell renal cell carcinoma. Nat Genet.

[39] Liu X lian (2022). PTEN loss confers sensitivity to rapalogs in clear cell renal cell carcinoma. Acta Pharmacol Sin.

[40] de Velasco G (2018). Targeted genomic landscape of metastases compared to primary tumours in clear cell metastatic renal cell carcinoma. Br J Cancer.

[41] Guo G (2012). Frequent mutations of genes encoding ubiquitin-mediated proteolysis pathway components in clear cell renal cell carcinoma. Nat Genet.

[42] Stransky LA (2022). Sensitivity of *VHL* mutant kidney cancers to HIF2 inhibitors does not require an intact p53 pathway. Proc Natl Acad Sci U S A.

[43] Li F (2021). Kidney cancer biomarkers and targets for therapeutics: survivin (BIRC5), XIAP, MCL-1, HIF1α, HIF2α, NRF2, MDM2, MDM4, p53, KRAS and AKT in renal cell carcinoma. J Exp Clin Cancer Res.

[44] Sekino Y (2020). PTEN is involved in sunitinib and sorafenib resistance in renal cell carcinoma. Anticancer Res.

[45] Takemura K (2023). Outcomes of patients with brain metastases from renal cell carcinoma treated with first-line therapies: results from the International Metastatic Renal Cell Carcinoma Database Consortium (IMDC). J Clin Oncol.

[46] Ho TH (2017). Differential gene expression profiling of matched primary renal cell carcinoma and metastases reveals upregulation of extracellular matrix genes. Ann Oncol.

[47] Barrett RL, Puré E (2020). Cancer-associated fibroblasts and their influence on tumor immunity and immunotherapy. Elife.

[48] Shani O (2021). Evolution of fibroblasts in the lung metastatic microenvironment is driven by stage-specific transcriptional plasticity. Elife.

[49] Chen Y (2021). Clinical and therapeutic relevance of cancer-associated fibroblasts. Nat Rev Clin Oncol.

[50] Shani O (2020). Fibroblast-derived IL33 facilitates breast cancer metastasis by modifying the immune microenvironment and driving type 2 immunity. Cancer Res.

[51] Callea M (2015). Differential expression of PD-L1 between primary and metastatic sites in clear-cell renal cell carcinoma. Cancer Immunol Res.

[52] Thompson RH (2004). Costimulatory B7-H1 in renal cell carcinoma patients: indicator of tumor aggressiveness and potential therapeutic target. Proc Natl Acad Sci U S A.

[53] Gulati S, Vogelzang NJ (2021). Biomarkers in renal cell carcinoma: are we there yet?. Asian J Urol.

[54] Zhu J (2018). Biomarkers of immunotherapy in urothelial and renal cell carcinoma: PD-L1, tumor mutational burden, and beyond. J Immunother Cancer.

[55] Steiert TA (2023). A critical spotlight on the paradigms of FFPE-DNA sequencing. Nucleic Acids Res.

[56] Lim AR, Ghajar CM (2022). Thorny ground, rocky soil: tissue-specific mechanisms of tumor dormancy and relapse. Semin Cancer Biol.

[57] Werner-Klein M (2020). Interleukin-6 trans-signaling is a candidate mechanism to drive progression of human DCCs during clinical latency. Nat Commun.

[58] Werner-Klein M (2018). Genetic alterations driving metastatic colony formation are acquired outside of the primary tumour in melanoma. Nat Commun.

[59] Hanahan D, Coussens LM (2012). Accessories to the crime: functions of cells recruited to the tumor microenvironment. Cancer Cell.

