# Supplementary material for: Molecular analysis of primary and metastatic sites in patients with renal cell carcinoma
Source: J Clin Invest. 2024 May 30;134(14):e176230. doi: 10.1172/JCI176230 (PMC11245151; doi:10.1172/JCI176230)

Supplementary Data

ACKNOWLEDGMENTS:

SG: supported by the National Cancer Institute of the National Institutes of Health under Award Number K08CA273542. The content is solely the responsibility of the authors and does not necessarily represent the official views of the National Institutes of Health.

CONFLICT OF INTEREST STATEMENT:

Shuchi Gulati: research funding from AstraZeneca (to institution). Advisory board fees for EMD Serono and AVEO. Honoraria from Mashup Media, MJH LifeSciences, MEC (Medical Educators Consortium), ASCO
Elliott Andrew: Employed by Caris
Arpit Rao: No disclosures
Benjamin Adam Gartrell: No disclosures
Chadi Nabhan: Employed by Caris and a shareholder
Charles J. Ryan: No relevant disclosures
Daniel Magee: Salaried employee at Caris Life Sciences with stock ownership. No advisory/management consulting positions aside from Caris. Patent US20210295979A1 – not relevant to this manuscript
Elizabeth Heath: Astellas funding; Arvinas: Research Funding; AstraZeneca: Research funding, Ad Board; Bayer: Honoraria, Ad Board; BioXcel Therapeutics: Research Funding Bristol-Myers Squibb: Research Funding; Calibr: Research Funding; Calithera Biosciences Inc.: Research Funding; Caris Life Sciences: Research Funding, Paid Travel; Corcept Therapeutics: Research Funding; Corvis Pharmaceuticals: Research Funding; Daiichi Sankyo Inc.: Research Funding; Eisai Inc.: Research Funding; Exelixis: Research Funding; Five Prime Therapeutics: Research Funding; Fortis Therapeutics: Research Funding; GlaxoSmithKline: Research Funding; Gilead Sciences Inc.: Research Funding; Harpoon Therapeutics: Research Funding; Hoffman-La Roche: Research Funding: Infinity Pharmaceuticals: Research Funding; iTeos Therapeutics: Research Funding: Janssen Research & Development LLC: Research Funding, Steering Committee; Merck Sharp & Dohme: Research Funding; Merck: Research Funding: Mirati Therapeutics: Research Funding; Modra Pharmaceuticals: Research Funding; Oncolys BioPharma: Research Funding; Peloton Therapeutics Inc.: Research Funding; Pfizer: Research Funding; Pharmacyclics LLC: Research Funding; POINT Biopharma: Research Funding; Sanofi: Honoraria, Speakers' Bureau, Paid Travel, Ad Board; Seattle Genetics: Honoraria, Paid Travel, Research funding
Earle Burgess: Stock Ownership: Exelixis, Becton Dickinson, Gilead Sciences, Medtronic
Honoraria: Exelixis, Janssen Oncology, Novartis, Pfizer, Merck
Research Funding: Pfizer, Astellas Pharma
James Brugarolas: has/had an advisory role for Arrowhead Pharmaceuticals, Eisai, Johnson and Johnson, Exelixis, Calithera, and Telix. In addition, he has patents for HIF2 biomarkers and mechanisms of resistance pending
Mathew Zibelman: Ad Boards: Exelixis, Imvax, EMD Serono, Jannsen;
Institutionally-Directed Research Funding: BMS, Exelixis
Mehmet Asim Bilen: Has acted as a paid consultant for and/or as a member of the advisory boards of Exelixis, Bayer, BMS, Eisai, Pfizer, AstraZeneca, Janssen, Calithera Biosciences,

Genomic Health, Nektar, EMD Serono, SeaGen, and Sanofi and has received grants to his institution from Merck, Xencor, Bayer, Bristol-Myers Squibb, Genentech/Roche, SeaGen, Incyte, Nektar, AstraZeneca, Tricon Pharmaceuticals, Genome & Company, AAA, Peloton Therapeutics, Ambrx, and Pfizer.
Nancy Dawson: No disclosures
Pedro Barata: Consultant: Astellas; AstraZeneca, AVEO Oncology, Bayer, BMS, Caris Life Sciences, Dendreon, Eisai, EMD Serono, Exelixis, Guardant Health, Janssen, Pfizer, Seattle Genetics; Research Grant: AstraZeneca, Caris Life Sciences, ESSA Pharma, Exelixis, Merck, Merus; Natera Nektar Therapeutics; Speaker's Bureau (Unbranded): AstraZeneca, Bayer, Caris Life Sciences, Sanofi, Pfizer Other: UroToday
Przemyslaw Twardowski: No disclosures
Rana McKay: Advisory Board/Consulting: AstraZeneca, Aveo, Bayer, Bristol-Myers Squibb, Calithera, Caris, Dendreon, Eli Lilly, Esiai, Exelixis, Janssen, Merck, Myovant, Novartis, Pfizer, Sanofi, SeaGen, Sorrento Therapeutics, Telix, Tempus. Institutional Research Support: AstraZeneca, Artera, Bayer, Bristol-Myers Squibb, Exelixis, Oncternal, Tempus.
Sourat Darabi: Consulting and advisory work: Oncolens, Bayer, BostonGene
Shuanzeng Wei is a board member of molecular tumor board of Caris Life Sciences
Tian Zhang: research funding from Acerta, Novartis, Merrimack, Abbvie/StemCentrx, Merck, Regeneron, Mirati Therapeutics, Janssen, Astra Zeneca, Pfizer, OmniSeq, Personal Genome Diagnostics, Astellas, Eli Lilly, Exelixis, BMS, Tempus; advisory board/consulting fees from Merck, Exelixis, Sanofi-Aventis, Janssen, Astra Zeneca, Pfizer, Amgen, BMS, Pharmacyclics, SeaGen, Calithera, QED Therapeutics, Eisai, Aveo, Eli Lilly, Aravive, Bayer, Caris; Honoraria from MJH Associates, Vaniam, Aptitude Health, Peerview, Clinical Care Options, KCA, SIU, and ASCO.
Toni K. Choueiri: reports institutional and/or personal, paid and/or unpaid support for research, advisory boards, consultancy, and/or honoraria past 5 years and ongoing, from:
Alkermes, AstraZeneca, Aravive, Aveo, Bayer, Bristol Myers-Squibb, Calithera, Circle Pharma, Eisai, EMD Serono, Exelixis, GlaxoSmithKline, Gilead, IQVA, Infinity, Ipsen, Jansen, Kanaph, Lilly, Merck, Nikang, Nuscan, Novartis, Oncohost, Pfizer, Roche, Sanofi/Aventis, Scholar Rock, Surface Oncology, Takeda, Tempest, Up-To-Date, CME events (Peerview, OncLive, MJH, CCO and others), outside the submitted work.
Institutional patents filed on molecular alterations and immunotherapy response/toxicity, and ctDNA.
Equity: Tempest, Pionyr, Osel, Precede Bio, CureResponse, InnDura.
Committees: NCCN, GU Steering Committee, ASCO/ESMO, ACCRU, KidneyCan.
Medical writing and editorial assistance support may have been funded by Communications companies in part.
No speaker's bureau.
Mentored several non-US citizens on research projects with potential funding (in part) from non-US sources/Foreign Components.
The institution (Dana-Farber Cancer Institute) may have received additional independent funding of drug companies or/and royalties potentially involved in research around the subject matter.
T. K. Choueiri is supported in part by the Dana-Farber/Harvard Cancer Center Kidney SPORE (2P50CA101942-16) and Program 5P30CA006516-56, the Kohlberg Chair at Harvard Medical School and the Trust Family, Michael Brigham, Pan Mass Challenge, Hinda and Arthur Marcus Fund and Loker Pinard Funds for Kidney Cancer Research at DFCI.

Supplementary Figure 1. Gene expression profiling of genes significantly upregulated in Figure 5A/B for each metastatic site relative to kidney among clear cell RCC only and non-clear cell only RCC cohorts.

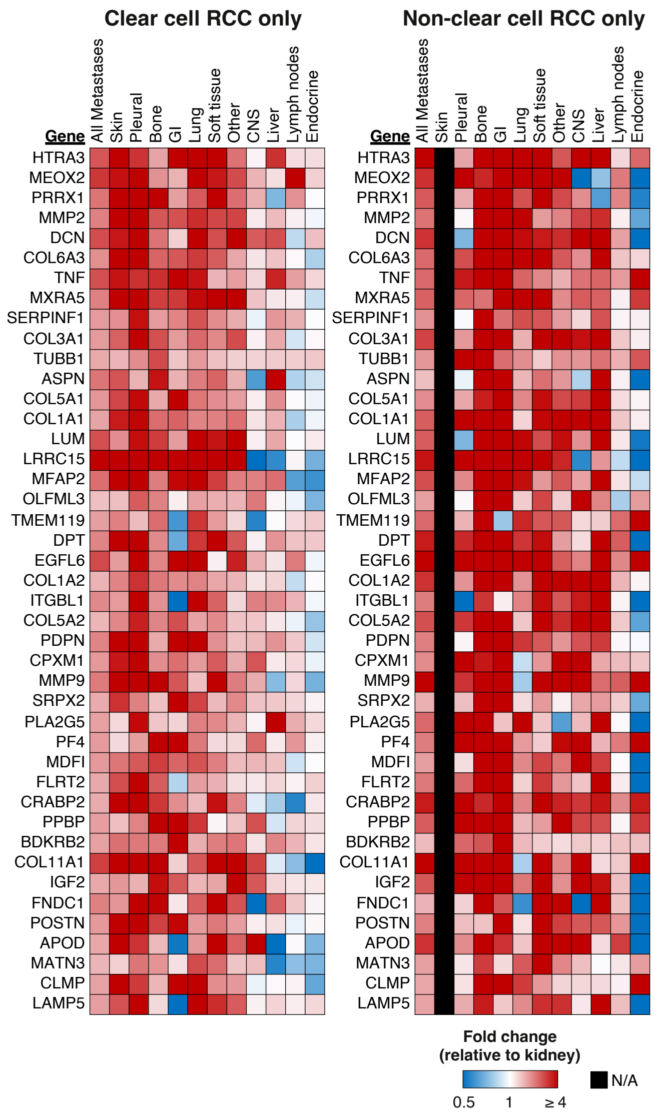

Supplement: Supplemental data [file jci-134-176230-s049.pdf]
